# Automatic Evaluation of Progression Angle and Fetal Head Station through Intrapartum Echographic Monitoring

**DOI:** 10.1155/2013/278978

**Published:** 2013-09-09

**Authors:** Sergio Casciaro, Francesco Conversano, Ernesto Casciaro, Giulia Soloperto, Emanuele Perrone, Gian Carlo Di Renzo, Antonio Perrone

**Affiliations:** ^1^National Research Council, Institute of Clinical Physiology, University Campus Ecotekne, Via Monteroni, 73100 Lecce, Italy; ^2^Department of Obstetrics and Gynecology, University of Perugia, Santa Maria della Misericordia University Hospital, San Sisto, 06132 Perugia, Italy; ^3^Obstetrics and Gynecology Department, “Vito Fazzi” Hospital, Piazza Filippo Muratore, 73100 Lecce, Italy

## Abstract

Labor progression is routinely assessed through transvaginal digital inspections, meaning that the clinical decisions taken during the most delicate phase of pregnancy are subjective and scarcely supported by technological devices. In response to such inadequacies, we combined intrapartum echographic acquisitions with advanced tracking algorithms in a new method for noninvasive, quantitative, and automatic monitoring of labor. Aim of this work is the preliminary clinical validation and accuracy evaluation of our automatic algorithm in assessing progression angle (PA) and fetal head station (FHS). A cohort of 10 parturients underwent conventional labor management, with additional translabial echographic examinations after each uterine contraction. PA and FHS were evaluated by our automatic algorithm on the acquired images. Additionally, an experienced clinical sonographer, blinded regarding the algorithm results, quantified on the same acquisitions of the two parameters through manual contouring, which were considered as the standard reference in the evaluation of automatic algorithm and routine method accuracies. The automatic algorithm (mean error ± 2SD) provided a global accuracy of 0.9 ± 4.0 mm for FHS and 4° ± 9° for PA, which is far above the diagnostic ability shown by the routine method, and therefore it resulted in a reliable method for earlier identification of abnormal labor patterns in support of clinical decisions.

## 1. Introduction

The monitoring of pregnancy demands for safe and accurate methods, tailored upon the specific gestational stage, is aiming to obtain a baseline evaluation of the anatomy and prenatal health of the fetus, ultimately trying to provide specific indications towards the best possible delivery management. Diagnostic imaging and a number of clinical tests (i.e., amniocentesis ande cordocentesis) are often used for the early pregnancy monitoring [1]. By this stage, clinical considerations can lead to the indication towards a Caesarian Section (CS) based upon fetal health and the physiology of the pregnant patient (e.g., previous CS, pelvis conformation, etc.). 

Successively, throughout the course of gestation, obstetrics and gynecologists are supported by few biomedical devices introduced in the last decades, such as intrapartum Electronic Fetal heart rate Monitoring (EFM) [2–4], external tocodynamometer, or internal intrauterine amniotic pressure sensor for uterine contraction monitoring [5, 6]. Furthermore, quantitative processing of electrohysterogram (EHG) data has experimentally proven its advantages over current practice in monitoring uterine contractile activity [7–11]; labor and nonlabor contraction classifications could enable the prediction of preterm delivery and, in case, the proper planning of an operative delivery [12, 13]. 

Therefore, the currently available methodologies allow the clinicians to formulate in advance clinical recommendations towards CS or operative childbirth in the minority of cases; for the remaining patients, no indicators to date have been found to identify the correct timing and modality of interventional childbirth. These parturients reach the final stage of pregnancy in jeopardy when entering the delivery room, since an incorrect management of childbirth labor may have a crucial impact on the neonatal health regardless of the cares taken during the course of the gestation. In fact, intrapartum assessment of progression indicators (cervical dilatation, fetal head station (FHS) and rotation, progression angle (PA), etc.), essential for deciding for a surgical (i.e., CS) or an operative intervention (i.e., application of forceps or vacuum extractor), is currently performed by highly subjective transvaginal manual inspections, although relevant literature extensively reported evidence of their unreliability with errors up to 88% in FHS [14] and up to 50% in cervix dilatation [15, 16] assessment. Using ultrasound (US) assessment as the standard reference, a high rate of error (65%) in transvaginal digital determination of fetal head position during the second stage of labor was also demonstrated, almost independently of the operator's experience [17–21]. The occurrence of these wrong assessments combined with the uncertain correlation between fetal distress and EFM [5] may cause uncontrollable fatal complications. The most concerning consequence of such lack of objective evidence in support of clinical choices, coupled with the inherently poor sensitivity and reliability problems of EFM [22], is the currently unacceptable rate of CS, largely above the 15% recommended by the World Health Organization [23]. 

Recently, various attempts have been made to design instruments for improving accuracy of cervical dilatation and fetal head station measurements [24–27], but none of them introduced significant advancement in the labor management. Being US the best suited method for safe and real-time childbirth-related diagnostic purposes, as it uses non-ionizing radiation and offers intraoperative guidance features [28–32], recently proposed diagnostic techniques for intrapartum monitoring involve transperineal US measurements of fetal head engagement [33], transvaginal sonographic assessment of the cervix [34], or positioning echographic receivers and electrodes pinned on the fetal head scalp [24]. These methodology are characterized by invasiveness and discomfort for the parturient as well as an increased infection risks for the fetus [35]. On the other hand, intrapartum translabial echographic acquisitions demonstrated their efficacy in imaging fetal head underneath the pubic bone [36]. Thus, we combined the latter modality with a pattern tracking algorithm, in order to automatically measure several labor progression parameters [37], realizing a new method for noninvasive, quantitative, and automatic monitoring of childbirth labor. Whereas a preliminary evaluation of the feasibility of the method in the clinical practice has already been conducted [38], this study represents the first quantitative comparative analysis of the outcomes of our algorithm with other methods. In particular, aim of this work is to perform a preliminary clinical validation of this new technique, quantifying its actual accuracy with respect to manual methods, representing the currently adopted “routine method,” and to a reference standard, represented by the echographic manual contouring performed by an experienced operator.

## 2. Materials and Methods

A US system was combined with a real-time tracking algorithm in order to automatically measure labor progression parameters, like fetal head station (FHS), head position, and progression angle (PA), based on patient specific anatomical references (Patent no. PCT/EP2009/008321) [37]. The algorithm was in-house developed (MatLab R2011b; The MathWorks Inc.) and employs a combination of morphological filters and pattern recognition methods [39] to perform the automatic segmentation and tracking of the fetal head outline and the pubic symphysis axis. 

A clinical digital echograph (MyLab70 XVG, Esaote Spa, Florence, Italy), employing a 2D convex ultrasound transducer (CA631, Esaote Spa, Florence, Italy), connected to a PC for real-time image processing was employed to measure FHS and PA (see description of the algorithm in Section 2.1). First, a validation study was carried out on a birth simulator (details of the experimental setup are provided in Section 2.2); then, a preliminary clinical validation (Section 2.3) was conducted on patients in labor using the developed method and the corresponding algorithm. Obtained results were compared with the current clinical routine method (i.e., transvaginal digital inspection) and the reference standard (i.e., image-based measurement of considered parameters by an experienced sonographer).

### 2.1. Description of the Algorithm

**Figure 1:**
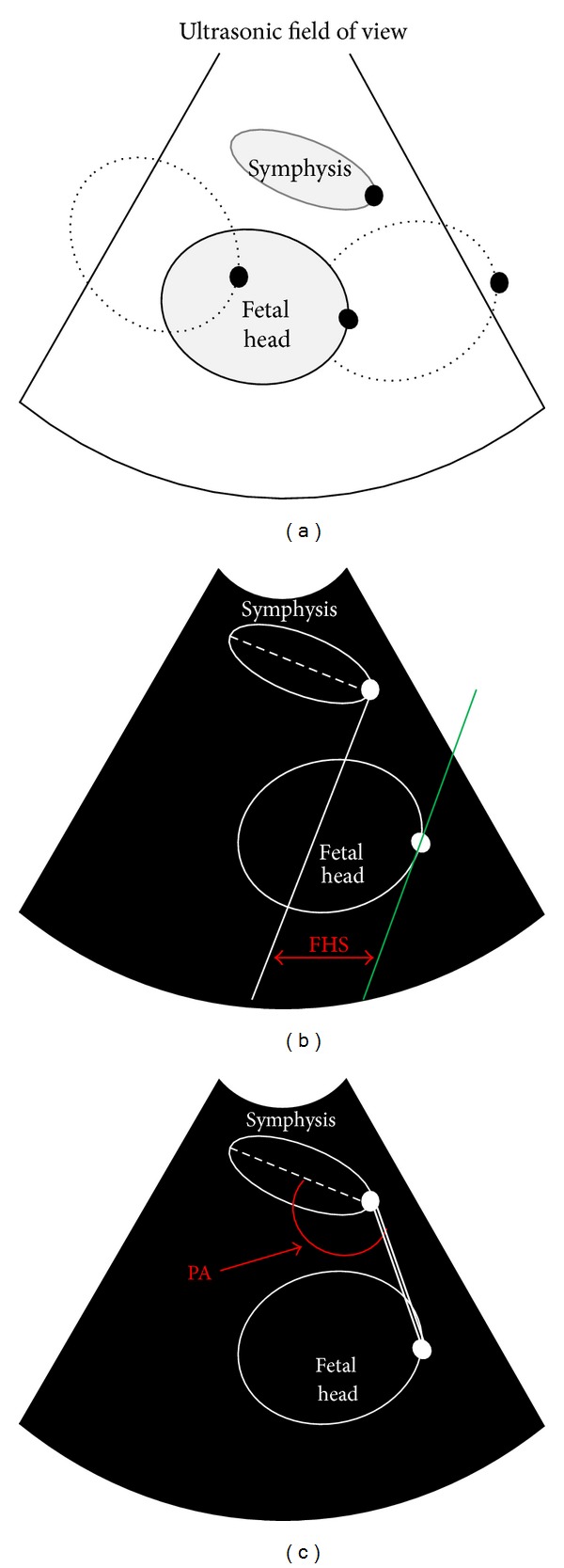
(a) Schematic diagram of the experimental setup adopted for assessment of tracking accuracy. (b) Scheme of the conventions is adopted to define fetal head station (FHS) indicated in red, where the solid white line is the line perpendicular to the symphysis longitudinal axis (dashed white line); the green line is the line passing by the fetal head outermost point; (c) scheme of the conventions adopted to define progression angle (PA) indicated in red, where the double white line is the connection between the symphysis distal end and the fetal head outermost point. By positioning the fetal head at different locations in the space comprised within the maternal pubic bone (birth canal), different stages of labor were simulated and relative position of the fetal head outermost point and distal end of the symphysis (both indicated by a solid black dot) were evaluated in terms of FHS and PA.

The method is used for automatic labor monitoring processes B-mode echographic image frames by means of the new algorithm, based on pattern tracking, for the calculation of FHS and PA along the typical trajectory of fetal head within the birth canal (Figure 1(a)).

In particular, FHS is defined as the horizontal distance between the line perpendicular to the symphysis longitudinal axis and a parallel line, passing by the fetal head outermost point (Figure 1(b)), whereas the PA is the angle comprised between the symphysis longitudinal axis and the line connecting the distal end of the symphysis with the fetal head outermost point (Figure 1(c)). 

**Figure 2:**
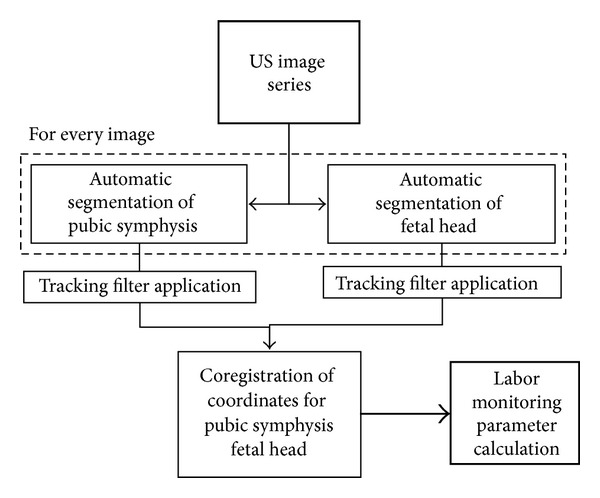
Schematic illustration of algorithm's working principle. The dashed line represents the iterative processing occurring on each image frame of the echographic acquisition.

The algorithm's working principle is schematically illustrated in Figure 2 and could be described as follows:

- each B-mode image is processed by the algorithm applying, separately, two dedicated sets of filters in order to selectively enhance the regions containing the fetal head outline and the symphysis medial axis;
- on the initial B-mode image analyzed, the two sub-structures are automatically segmented and identified as the two patterns to be searched within the subsequent images by means of maximization of either similarity or crosscorrelation coefficients [39];
- pubic symphysis axis and distal end are segmented on subsequent images and displacements from previous position are also calculated. Specifically, at the point corresponding to the distal end of the symphysis, a line perpendicular to the axis is defined;
- pattern location of fetal head is employed to initialize the automatic edge outlining from subsequent images and to calculate the displacement of fetal head rightmost point (i.e., the outermost point, when assuming the fetal descendent progresses from left to right) from previous position. A line is defined, passing by the fetal head outermost point and parallel to the line perpendicular to the symphysis axis;
- for each frame, coordinates and displacements of the fetal head are registered with respect to the reference system associated to the pubic symphysis distal end in order to calculate FHS and PA.

Obtained values were compared with the respective reference; furthermore, FHS measurements were also converted in one of the 11 possible stations (−5 cm to +5 cm distance from the plane of the ischial spines, which are slightly above the distal level of the symphysis), according to the definitions of the American College of Obstetricians and Gynaecologists (ACOG) [40].

### 2.2. Birth Simulator Experiments

An experimental setup was developed to reproduce the expected working conditions of a US probe adherent to the pubic area of the body of a parturient. 

The birth simulator was a mechanical device consisting of a maternal mannequin and a fetal head, reproducing anatomical features of pubic bone and fetal head in tissue-mimicking materials taking into account recently reported findings available in the literature [41–44]. The birth simulator was immersed in a water bath, in order to eliminate air within the different simulator components. The fetal head was moved along its typical trajectory within the birth canal, and the position of its outermost point was identified with respect to the distal end of the symphysis. Once the probe was fixed on the model of the symphysis, anatomical reference points were chosen by an operator on the initial echographic image; then, the fetal mannequin was moved following a pre-established sequence of locations, including all possible values of FHS (according to the corresponding definitions provided by the ACOG), PA, and several combinations of possible occiput presentations (anterior-posterior, left-right, etc.).

B-mode echographic image frames were acquired during the described experiments and were real time processed by the algorithm for the calculation of labor monitoring parameters.

### 2.3. Clinical Validation Study

A total of 10 parturients were recruited for this study employing the following criteria: singleton cephalic-presenting fetus, body mass index (BMI) <30 kg/m^2^, gestation age >38 weeks, absence of documented fetal malformations, active labor stage with cervical dilatation <2 cm, and informed consent. These selection criteria allow to evaluate the algorithm's performance in monitoring childbirth progression throughout all labor phases in nonobese women without biases of parameter calculation deriving from fetal malformations or severe dystocia. 

All the enrolled patients underwent the conventional labor management (continuous EFM and tocodynamometer, obstetric examinations, etc.) and an additional translabial echographic examinations, with prior application of ultrasonic coupling gel (Aquasonic 100, Parker Laboratories, Fairfield, NJ, USA) on the probe contact surface in order to eliminate air within the probe and the patient. The echographic acquisition was regularly performed, employing the same echographic system adopted for the birth simulator experiments, within 1 minute after the peak intensity of every contraction, as identified in the chart of the tocodynamometer connected to the parturient. 

Acquired B-mode US images were analyzed offline, in order to avoid interference with the normal clinical activity of the obstetrics unit, by our fully automatic custom-developed algorithm for image processing and pattern tracking, which provided the temporal evolution of PA and FHS.

The same parameters were also calculated upon manual contouring of the same images by an experienced operator (standard reference), that was blinded regarding the automatic algorithm results. The accuracy of algorithm results was quantified with respect to standard measurements for both FHS and PA. Obtained values were compared with the corresponding routine transvaginal measurements performed by experienced gynecologists at the same time instants during childbirth labor (routine method).

## 3. Results

In the birth simulator, the automatic identification of symphysis distal end and fetal head outermost point was correct in 98% of the computed images providing high visual reliability for the operator and the average errors (expressed as mean error ± 2SD) were 0.8 ± 1.8 mm for FHS and 3° ± 4° for the PA. Our results were achieved through maximization of the similarity coefficient at a frame rate that guarantee real-time monitoring of labor progression. However, accuracies of pattern tracking could be improved by about 30% through the maximization of the correlation coefficient, despite determining higher computational costs and a consequently lower frame-rate to be processed, that is, from 1 fps to 0.2 fps, which is still suitable for the purpose.

The methodology has been successfully translated in a preliminary intrapartum echographic study, during which the outcome of the routine method for FHS evaluation through digital inspection was conducted according to existing clinical protocols and recorded for our analyses.

**Figure 3:**
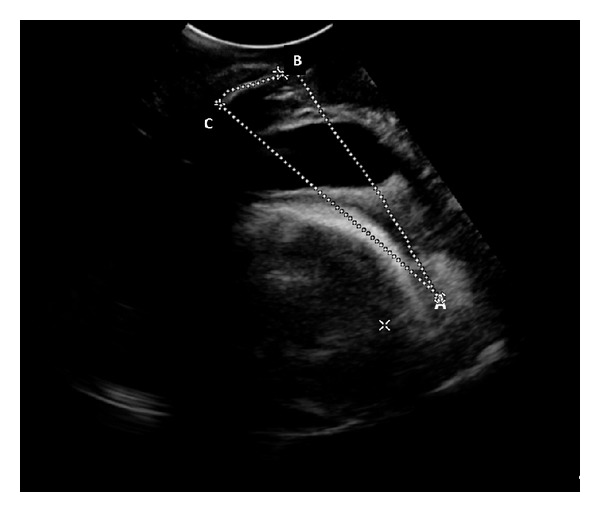
Example of manual contouring performed by an experienced sonographer (standard reference). “A” represents the fetal head outermost point; “B” and “C” are, respectively, the distal and proximal ends of the pubic symphysis.

Echographic imaging was performed immediately after contraction; pubic symphysis appeared always recognizable in the acquired US images whereas fetal head outline appeared sometimes discontinuous because portions of the US were attenuated by the pubic bone. In these cases the ultrasonic probe was not entirely positioned in correspondence with the cartilaginous pubic symphysis which would have, otherwise, allowed US transmission without attenuation. Nonetheless, the expert operator was able to manually detect on screen the references of the fetal head outermost point and the longitudinal axis of the symphysis (Figure 3) in order to elaborate their coordinates and calculate the PA and FHS values used as standard reference measurements. 

**Figure 4:**
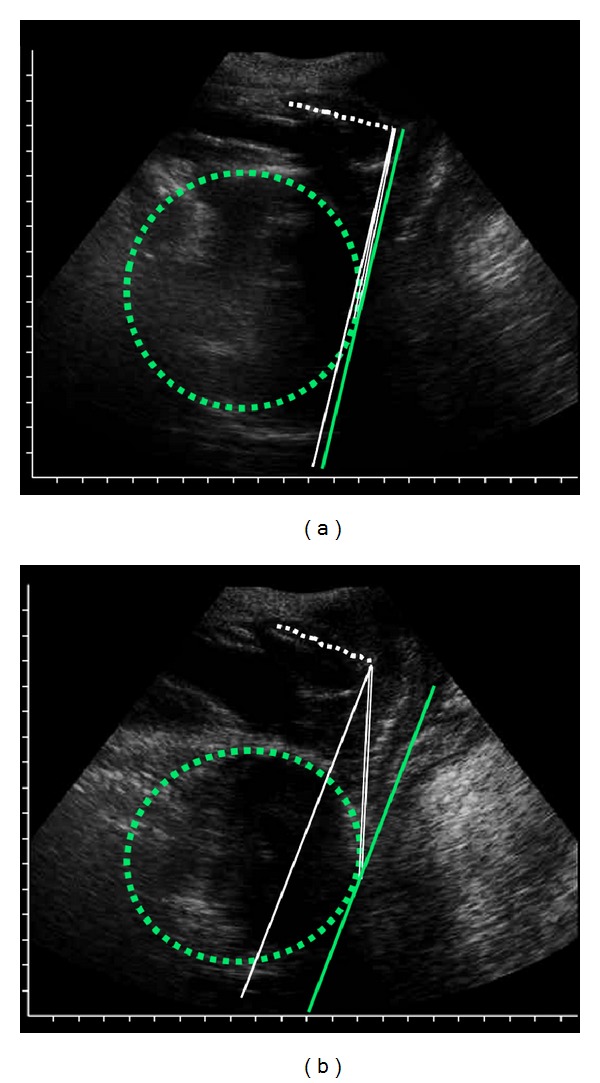
Example of algorithm output. The dashed green outline represents the fetal head; the white dashed line represents the symphysis longitudinal axis; the solid white line is the line perpendicular to the symphysis longitudinal axis; the solid green line is the line passing by the fetal head outermost point; the solid white double line is the connection between the symphysis distal end and the fetal head outermost point. (a) Image representing FHS = 0 cm and PA = 90°; (b) image representing FHS = 1.8 cm and PA = 103°.

In all the examined images, the tracking algorithm easily identified the symphysis, successfully interpolated fetal head outline and derived outermost point coordinates as well as the location of the distal end of the symphysis, as demonstrated by the results obtained on a 32-year-old woman at the 38th week of gestation of a 3.45 kg weighting baby boy, belonging to our cohort of patients (Figure 4). Results regarding this patient, taken as a typical case, will be further discussed in detail in this section to compare the accuracies of manual inspections versus the automatic calculations.

**Figure 5:**
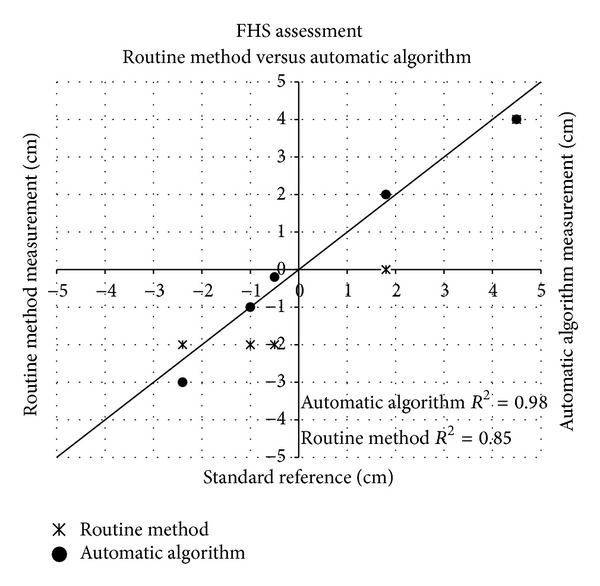
Scatter plot showing the fetal head station (FHS) measurements and corresponding automatic algorithm values, performed on a 32-year-old parturient at the 38th week of gestation, obtained through transvaginal manual inspection against the measurements obtained from the expert operator (standard reference), provided with the respective *R* ^2^. The line of equality is also shown.

FHS values obtained with the routine method and with the automatic algorithm (i.e., the FHS values calculated by the algorithm from those US images acquired approximately at the same time of digital inspection) were evaluated against the standard reference measurements. Specifically, due to its invasiveness, the routine method was executed on average more than 3 times less often than the translabial echographic acquisition. In addition to that, intrapartum measurements based on the routine method failed in addressing timely the different phases of labor progression identifying the correct ACOG station only in 20% of cases. The clinical consequence of a wrong assessment of the stage of labor in terms of ACOG station is potentially dangerous when a “nonengaged” head was misdiagnosed as “engaged”, since the possibly required maneuvers could be erroneously directed and, for instance, the inappropriate modality of forceps or vacuum application could be applied. The comparison between the routine method and the automatic algorithm measurements is presented in detail for the case chosen from the cohort of patients. We compared the measurements obtained after manual inspection of the birth canal with values of FHS calculated from echographic images acquired after the same contraction. Due to the invasiveness of the procedure, the number of manual inspections was in all cases smaller than the number of acquired echographic image sequences; for the considered patients, the clinical staff performed 5 vaginal inspections whereas the echographic image series acquired were 20. Therefore, only a portion of the automatic algorithm measurements was plotted in Figure 5. Within the limited number of values available for comparison, the FHS assessment performed through the automatic algorithm showed a high rate of agreement with the standard reference (*R* ^2^ = 0.98, *P* < 0.01), and in one case the measured value overlapped the line of equality, whereas employment of the routine method achieved a good yet lower agreement with the standard reference (*R* ^2^ = 0.85, *P* < 0.05) and led to missing the identification of the FHS value equal to “0” (Figure 5). 

**Figure 6:**
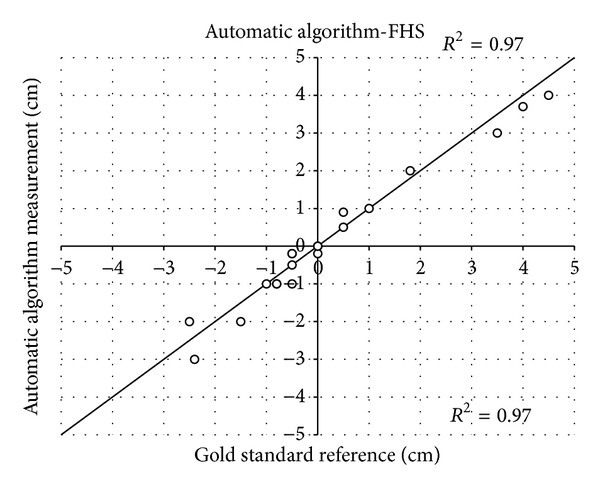
Scatter plot showing the fetal head station (FHS) measurements, performed on a 32-year-old parturient at the 38th week of gestation, obtained through the automatic algorithm against the measurements obtained from the expert operator (standard reference), provided with the respective *R* ^2^. The line of equality is also shown. In the stage of labor between stations “−1” and “0”, 3 points out of 20 are overlapped due to the lack of progression in consecutive FHS measurements.

**Figure 7:**
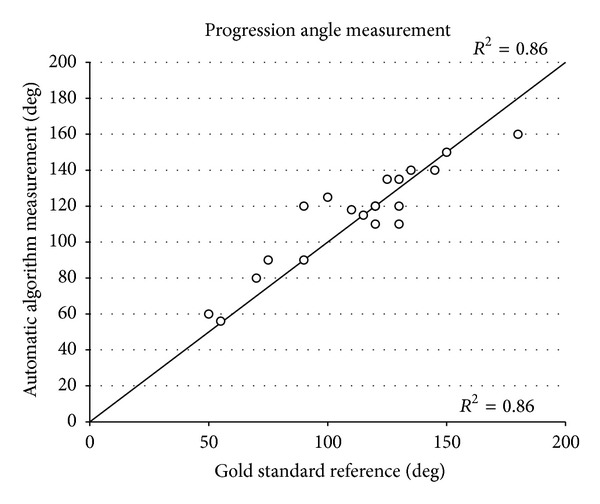
Scatter plot showing the progression angle (PA) measurements, performed on a 32-year-old parturient at the 38th week of gestation, obtained through the automatic algorithm against the measurements obtained from the expert operator (standard reference), provided with the respective *R* ^2^. The line of equality is also shown. At the stage of labor corresponding station “−1” and “0”, 1 point out of 20 is overlapped due to the lack of progression in consecutive PA measurements.

Moreover, referring to the same parturient, the 20 automatic measurement values of FHS performed throughout labor duration maintained high correlation (*R* ^2^ = 0.97, *P* < 0.001) with the standard reference, and this two techniques simultaneously identified the FHS value equal to “0” (Figure 6). Whereas PA is not currently assessed by manual inspection, its value throughout labor was measured by means of the standard reference methodology and the examined automatic algorithm. When compared, the two techniques demonstrated a good agreement (*R* ^2^ = 0.86, *P* < 0.001) although a minor overestimation of the parameter was shown by the automatic algorithm (Figure 7) over the 20 values examined. 

The evaluation of the two labor progression parameters measured by the different assessment methodologies was performed on the entire cohort of patients, returning global accuracy of automatic parameter measurement, compared to standard reference, of 0.9 ± 4.0 mm for FHS and 4° ± 9° for PA (mean error ± 2SD); thus automatically measured FHS values always are coincided to the correct ACOG station. 

## 4. Discussion

Our methodology, tested for labor monitoring on a number of volunteers, resulted well tolerated by the patients and allowed objective quantification of labor progression with a level of accuracy and time effectiveness higher than that achievable applying vaginal inspections (routine method). Similar to the performance assessed on the birth simulator, the algorithm successfully and timely identified, in the studied parturients, the correct ACOG stations also thanks to a number of measurements higher than those made with the routine method. Specifically, the FHS position “0” was correctly identified on US images, shown in Figure 4(a), by both automatic algorithm and standard reference method; conversely the manual inspection of the birth canal assessed such FHS level only 25 minutes later, simultaneously with the acquisition of the frame presented in Figure 4(b), when the FHS was already nearly level “+2”. Therefore, evaluation of labor progression made with the routine method was confirmed qualitative and subjective, implying, in some cases, that the fetus would stay in the same position within the birth canal for long time without knowledge of the event by the operators, enhancing the risk of fetal distress. In fact, the routine method presented potentially dangerous errors in 15% of measurements over the cohort of patients; defining dangerous errors of all those cases in which a “nonengaged” head misdiagnosed as “engaged” (i.e., FHS > −2 [40]) could lead to unnecessary operative interventions. Furthemore, the presented method provided high correlation with the reference gold standard in assessing both FHS and PA (*R* ^2^ = 0.97 and *R* ^2^ = 0.86, resp., both with *P* < 0.001), successfully monitoring the fetal head descendent. 

Future studies will include the repetition of experiments, similar to those reported in this work, employing a 3D ultrasound probe, whose field of view will allow the simultaneous measurement of FHS, PA, and, possibly, of other labor parameters, that were not considered in this study, that is, fetal head rotation; increased number of variable parameters measured by the algorithm would significantly improve operator's capability of assessing childbirth labor progression. 

Therefore, our approach can address the needs of evidence to support medical decision with quantitative, objective, and storable indicators in all those cases of unpredicted dystocic labor and in those cases in which indicator of dystocia predictors, obtained during pregnancy monitoring, failed to describe the actual scenario in the delivery room. The proposed methodology showed the ability to overcome the limits of current labor-monitoring methods, providing a possible effective tool for earlier identification of abnormal labor patterns and accurate decision-taking support. 

## 5. Conclusions

The study demonstrated the effectiveness of using ultrasound methods and automatic tracking algorithms for monitoring of labor progress. Specifically, the experiments conducted on the birth simulator were useful to quantify the accuracy of the newly developed method. Moreover, the clinical translation of the methodology was confirmed to be feasible through clinical validation on ten parturients, who did not manifest any kind of discomfort during the echographic examination. The implementation of the method allowed measuring labor progression indicators, such as FHS and PA, during all phases of labor with satisfactory accuracy compared to standard reference (0.9 ± 4.0 mm for FHS and 4° ± 9° for PA). 

Therefore, this new technique is qualified as an objective approach to childbirth labor monitoring and could provide additional and quantitative information throughout all phases of labor, potentially advancing the current clinical practice which merely relies on transvaginal digital inspections. In particular, compared to other quantitative experimental methods, our approach minimizes invasiveness for mother and babies. Furthermore, the automatic algorithms evaluated in this work could address the needs of new standardized quantitative monitoring approaches and new guidelines to possibly reduce the high rate of CS, and also providing documentation records of objective parameters to avoid legal litigations in case of damages to patients and/or to babies that occurred during delivery.
